# Sociodemographic, Clinical, and Therapeutic Characterization of Multiple Myeloma Patients (CharisMMa Study) with Symptomatic Relapse and/or Refractory Disease: An Observational, Multicenter Study in Portugal

**DOI:** 10.3390/hematolrep18030034

**Published:** 2026-05-19

**Authors:** Rui Bergantim, José Guilherme Freitas, Cristina Gonçalves, Helena Martins, Herlander Marques, Henrique Coelho, Patrícia Seabra, Adriana Roque, Márcio Tavares, Pedro Pinto, Ana Rita Francisco, Joana Tato, Catarina Geraldes

**Affiliations:** 1 i3S-Instituto de Investigação e Inovação em Saúde, University of Porto, 4200-135 Porto, Portugal; 2 Cancer Drug Resistance Group, IPATIMUP-Institute of Molecular Pathology and Immunology, University of Porto, 4200-135 Porto, Portugal; 3 Clinical Hematology, Unidade Local de Saúde de São João, E.P.E-Hospital Center of São João, 4200-319 Porto, Portugal; 4 Clinical Hematology, FMUP-Faculty of Medicine, University of Porto, 4200-319 Porto, Portugal; 5 Serviço Onco-Hematologia, Instituto Português Oncologia Porto, 4200-072 Porto, Portugal; 6 Unidade Local de Saúde de Santo António, E.P.E.-Serviço de Hematologia, Centro Hospitalar Universitário de Santo António, 4099-001 Porto, Portugal; 7 ICBAS-School of Medicine and Biomedical Sciences, University of Porto, 4050-313 Porto, Portugal; 8 Clinical Hematolology Unit, Unidade Local de Saúde Santa Maria, Hospital de Santa Maria, 1649-028 Lisboa, Portugal; 9 Faculty of Medicine, University of Lisboa, 1649-004 Lisboa, Portugal; 10 Unidade Local de Saúde de Braga, Serviço de Oncologia do Hospital de Braga, 4710-243 Braga, Portugal; 11 Centro Clínico Académico, de Braga-2CA-Braga, 4710-243 Braga, Portugal; 12 CINTESIS-Centro de Investigação em Tecnologias e Serviços de Saúde, 4200-450 Porto, Portugal; 13 Unidade Local de Saúde Gaia e Espinho, Clinical Hematology, Gaia Hospital, 4434-502 Porto, Portugal; 14 Clinical Hematology, Unidade Local de Saúde de Coimbra, 3000-075 Coimbra, Portugal; 15 Institute of Physiology, Faculty of Medicine, University of Coimbra, 3004-531 Coimbra, Portugal; 16 Unidade Local de Saúde Gaia e Espinho, Clinical Hematology, Centro Hospitalar Vila Nova de Gaia, 4430-062, Espinho, Portugal; 17 Takeda Farmacêuticos Portugal, 2770-071 Paço de Arcos, Portugal; 18 Laboratório de Oncobiologia e Hematologia e Clínica Universitária de Hematologia, Faculdade de Medicina, Universidade de Coimbra, 3004-531 Coimbra, Portugal; 19 Coimbra Institute for Clinical and Biomedical Research (iCBR), Grupo de Investigação em Ambiente, Genética e Oncobiogia (CIMAGO), Faculdade de Medicina, Universidade de Coimbra, e Centro de Inovação em Biomedicina e Biotecnologia (CIBB), 3004-531 Coimbra, Portugal; 20 Centro Académico-Clínico de Coimbra (CACC), 24230-322 Coimbra, Portugal

**Keywords:** multiple myeloma, relapse and/or refractory multiple myeloma, sociodemographic, clinical features, treatment, real-world data, comorbidities, risk factors

## Abstract

Objectives: Real-world information on relapsed and/or refractory multiple myeloma (rrMM) clinical management in Portugal is scarce. The CharisMMa Portugal study aimed to characterize rrMM patients through socio-demographic and clinical parameters and describe treatment patterns. Methods: This was an observational, cross-sectional, multicenter study with 62 rrMM patients routinely treated at seven hospitals in Portugal. Data were collected from medical records during clinical appointments (2020–2022) after written informed consent was obtained (ClincialTrials.gov ID-NCT04135963). Patients who were diagnosed with a symptomatic MM episode in the 6 months prior to study initiation and who received treatment before their last relapse episode were enrolled. Results: Most patients were male (54.8%) and living with relatives (90.3%), and almost 50% were independent. Roughly 70% of patients were classified as Revised MM International Staging System (R-ISS) Stage II at diagnosis, with a mean age of 65.76 (±9.24) years old. Most common SliM-CRAB (SLIM: sixty percent or more clonal plasma cells in the bone marrow (S), light chain ratio ≥100 (Li), and MRI-detected focal lesions (M); CRAB: hypercalcemia (C), renal insufficiency (R), anemia (A), and bone lesions (B)) signs were bone lesions (59%), and 62.9% of the patients had at least one comorbidity. At study initiation, 70.5% of patients were on second-line treatment, with monoclonal antibodies and proteasome inhibitors (PIs) + immunomodulators (IMiDs) as leading agents. Triplet regimens were the most common across all lines, while oral and oral + subcutaneous were the most prevalent routes of administration. Conclusions: Triple treatment combinations are common in rrMM management, with PIs and IMiDs frequently used, especially in first-line settings. The use of oral formulation is substantial, suggesting a step toward more patient-centric options. This characterization underscores the complexity of rrMM management and should inform stakeholders of strategies to standardize patient care across reference centers in Portugal.

## 1. Introduction

Multiple myeloma (MM) is a plasma cell neoplasm, characterized by various and potentially severe complications, and is the second most common hematological cancer, accounting for about 13% of all cases. In 2020, 176,404 new MM cases were reported in Europe, with a 5-year prevalence of 138,083 and 32,495 deaths [1]. In Portugal, MM incidence is around 7.8/100,000 [2].

The median age at diagnosis is 70 years [3], slightly above the median age in Portuguese data (69 years) [2]. As life expectancy increases, so does the incidence of MM [4]. This presents a significant challenge in countries with marked inverted demographic pyramid such as Portugal, a trend that is anticipated to become more striking as the birthrate is well below the necessary population replacement rates.

The MM patient journey often contemplates alternating cycles of treatment, periods of remission, and relapse events. Due to its biological heterogeneity, relapses manifest throughout the natural history of the disease, each exhibiting specific characteristics and distinct implications [5]. Relapsed and/or refractory MM (rrMM) translates into significant physical and psychological burden on patients and families, especially in older and frailer populations. Data from Portugal show that burden to caregivers increases with successive treatment lines [6]. Physicians face a substantial burden navigating the complexity of addressing relapses. This challenge entails considering not only the characteristics of the relapse and the patient but also social and familiar backgrounds, despite the availability of the numerous current and innovative therapies [5,7,8,9].

The emergence of a wide array of novel MM treatments [10], including immunomodulators (IMiDS), monoclonal antibodies (mAbs), and proteasome inhibitors (PIs), broadened the therapeutic arsenal beyond the conventional options—chemotherapy, stem cell transplantation, and supportive care—translating into better survival outcomes and improved quality of life [11,12]. More recently, bispecific antibodies [13,14], CAR T cells [15,16] or even antibody–drug conjugates (ADCs), such as belantamab mafodotin [17,18,19] have proven the added potential benefits of novel therapeutics in improving the clinical outcomes in MM patients. Regardless of the outstanding therapeutic advances, the heterogeneity of MM implies high variability in both treatment response and survival outcomes [20]. Therefore, this patient population requires more effective, well-tolerated, and personalized therapeutic strategies to achieve high-quality responses and overcome this heterogeneous and challenging hematologic malignancy [21].

In Portugal, access to care in oncology centers is primarily organized within the National Health Service (SNS), which ensures universal coverage. However, specific criteria and structured systems help determine which patients are eligible for care in these centers. Patients typically access cancer centers through referrals from primary care physicians or specialists. The referral is based on confirmed diagnoses or suspicion of cancer. The National Oncology Referral Network outlines guidelines for referring patients to specialized cancer centers, focusing on the complexity and type of cancer, ensuring appropriate treatment in centers of excellence. On the other hand, access may depend on clinical factors, such as the stage of the disease, comorbidities, and the potential to benefit from specific innovative therapies or clinical trials. While the SNS strives for equitable access, the geographical location of the patient relative to a cancer center may influence access, although transport assistance may be available if required. Information in the country regarding the rrMM population and its clinical management in the real-world setting is scarce. The objective of the CharisMMa Portugal study was to characterize rrMM patients regarding socio-demographic and clinical features, as well as to describe treatment patterns.

## 2. Materials and Methods

### 2.1. Study Design and Population

This was an observational, cross-sectional, multicenter study using a consecutive sample of rrMM patients routinely managed at seven reference public hospitals in Portugal. For reasons of transparency, the CharisMMa Portugal study was registered on the ClinicalTrials.gov platform (ID–NCT04135963) on 21 October 2019.

The study population consisted of patients with an rrMM diagnosis established within the 5 years before study inclusion and who had received at least one previous line of treatment. This timeframe was selected to reduce variability due to evolving treatment protocols, ensuring a more up-to-date characterization. Additionally, patients must have started a new treatment line due to a relapsed/refractory disease in the 6 months prior to study inclusion. Data were collected from medical charts and through patient interviews during routine clinical appointments between July 2020 and September 2022. Symptomatic relapse was considered a recurrence of the signs and symptoms of MM, such as anemia, renal failure, and/or bone pain, as stated in the International Myeloma Working Group (IMWG) guidelines [22]. In this study, symptomatic recurrence in rrMM was defined as the reappearance or progression of disease-related clinical manifestations, which can include anemia, bone lesions, renal dysfunction, hypercalcemia, or other organ damage, as outlined by the IMWG criteria, occurring after a period of response or stability following prior therapy.

All patients provided written informed consent. Each local independent ethics committee approved the study protocol before the start of data collection.

### 2.2. Study Data

Patient socio-demographic information included sex, body mass index (BMI), area of residence, educational level, employment and cohabitation status, financial assistance need, degree of dependence [23], physical activity level, smoking status, and alcohol use, which were all recorded during the study appointment.

Clinical data for patients at the time of MM diagnosis included age, staging according to the International Staging System (ISS) and Revised ISS (R-ISS), and ECOG performance status. Regarding the latest rrMM episode, SLiM-CRAB criteria, cytogenetic analysis and risk-strata based on cytogenetic profile (mSMART v2 and mSMART v3), clinical presentation, comorbidities, and paraprotein characteristics were collected.

Furthermore, treatments previously prescribed and those ongoing during study appointments were recorded.

### 2.3. Statistical Analysis

Sample size was calculated to allow for a robust characterization of rrMM patients in the Portuguese setting. From the available data for Portugal, assuming a confidence level of 95% and a margin of error of 7.5%, the calculated sample size was 151 rrMM patients. However, due to recruitment constraints, 74 patients were screened and only 62 patients were analyzed.

All eligible patients were included in the full-analysis set (FAS). Data were summarized using descriptive statistics. Statistical analyses were carried out with SAS^®^ software (version 9.4; SAS Institute Inc., Cary, NC, USA) [24].

## 3. Results

### 3.1. Patient Disposition and Sociodemographic Characterization

**Figure 1:**
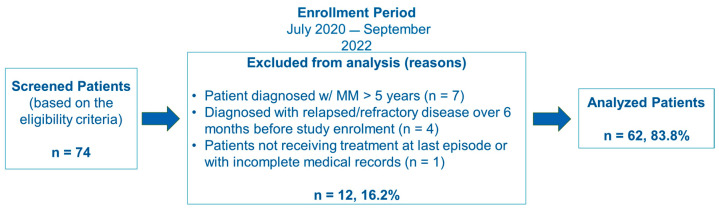
Patient disposition.

Of the 74 patients who were screened, 62 patients (83.8%) were included in the FAS. The reasons for screening failure are detailed in Figure 1. 

Most patients were male (54.8%) (Table 1). The mean BMI was 25.48 kg/m2 (±3.86) with overweight/obesity found in nearly 55% of patients. Most patients (67.7%) resided in urban areas, had a primary education level (65.5%), and were retired (62.3%). Almost all patients (90.3%) lived with relatives, 49.2% were independent, and 18.3% were in need of financial assistance. Regarding lifestyle factors, over half of the patients (56.7%) practiced low to moderate physical activity, 11.3% were current smokers, and 8.1% reported alcohol consumption.

### 3.2. Clinical Characterization

The mean age at MM diagnosis was 65.76 (±9.24) years old, and the mean time from the last relapse to the study visit date was 2.57 months (±1.76). Regarding MM staging, 69.6% of patients were classified as R-ISS Stage II at diagnosis and 37.1% were categorized as stage II using the ISS staging system (Table 2). At the time of MM diagnosis, 5.1% of patients had an ECOG-PS 3 performance status, with most patients (74.6%) reporting ECOG-PS 0–1. At diagnosis, 80% of patients presented with a Heavy Chain type. Among these, 56.7% had an IgG Heavy Chain, while 21.7% had an IgA Heavy Chain.

The most common SLiM-CRAB signs at last relapse were bone lesions (59.0% of patients), followed by anemia (45.9%).

Among the 24 patients who underwent cytogenetic analysis at the last relapse, 11 (45.8%) reported at least one cytogenetic abnormality. Of these eleven patients, eight (72.7%) were classified as high-risk.

Bone fractures were identified in 22.6% of the patients at last relapse. Medullary/extramedullary plasmacytomas were present in 64.9% of patients, with 52.6% reporting two or more plasmacytomas.

At last disease relapse, the average LDH level was 233 (±169) IU/L, with 26.6% of patients presenting with elevated LDH levels (>250 IU/L). The mean serum paraprotein level was 53.19 (±105.42) g/L. The mean serum kappa free light chain (FLC) level was 42.08 (±123.02) g/L. The mean serum lambda FLC level was 16.08 (±31.81) g/L.

In the last rrMM episode, 62.9% had at least one concomitant disease, with the most prevalent being diabetes (22.6%, n = 14), cardiovascular disease (17.7%), and peripheral neuropathy (16.1%). Around 42% (41.9%) had another secondary disorder.

### 3.3. Treatment Patterns

#### 3.3.1. Before Last Relapse

**Figure 2:**
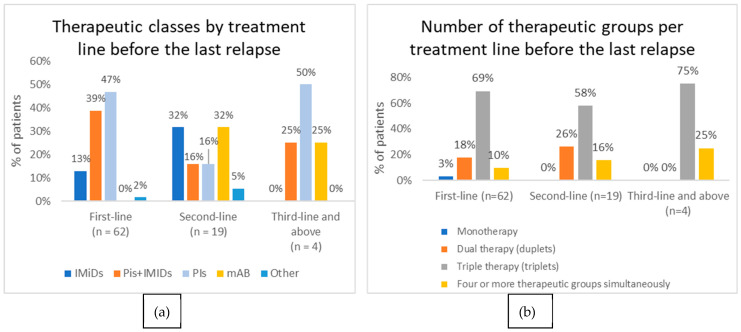
IMiDs = immunomodulatory drugs. PIs = proteasome inhibitors. mAbs = monoclonal antibodies. (**a**) Distribution of prescribed therapeutic class before last relapse by treatment line; (**b**) distribution of treatment combinations before last relapse by treatment line.

The median treatment duration before the last relapse was 165.50 days (IQR: 112.5–279.0). In total, 19 patients (30.6%) received second-line treatment before their last relapse preceding the study visit, while four patients (6.5%) received third-line or above treatment. PIs were the agents most used in first-line treatment (46.8% of patients), while a combination of PI and IMiDs was used in 38.7% of patients. mAbs became more common during second-line treatment, accounting for 31.6% of patients. In third-line and above treatments, PIs re-emerged as the most frequently used option (50.0% of patients), either retreatment with the same PI or a different PI (Figure 2a). The rate of transplants was 24.2% in first-line treatment (15 out of 62 patients), decreasing to 15.8% in second-line treatment (3 out of 19 patients).

Triplet regimens were predominant across all treatment lines, accounting for 69.4%, 57.9% and 75% of cases in first-, second- and third/subsequent-line treatments, respectively (Figure 2b).

**Figure 3:**
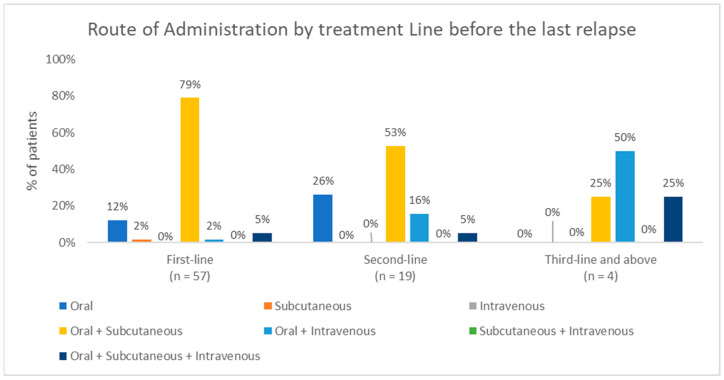
Distribution of routes of administration prescribed before the last relapse by treatment line.

Oral plus subcutaneous were the main routes of administration during first-line treatment (78.9% of patients). In third-line and subsequent treatments, combined oral and intravenous administration became the predominant approach (50%). Additionally, the oral route was used exclusively for 12.3% during first-line treatment and for 26.3% of patients during second-line treatment (Figure 3).

**Figure 4:**
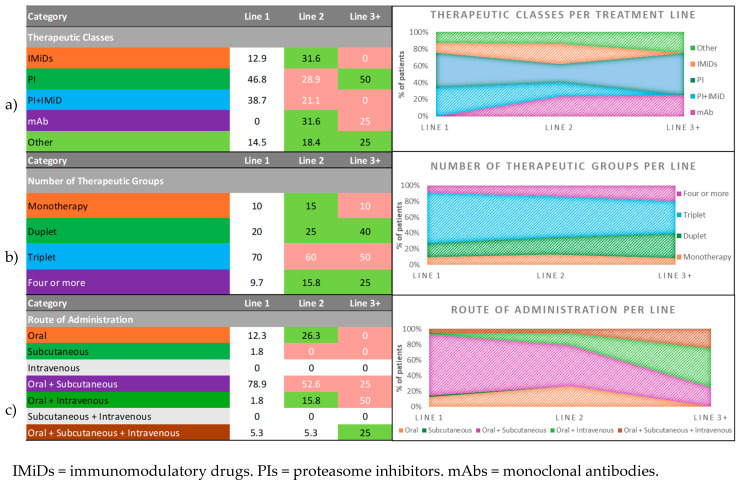
(**a**) Distribution of therapeutic classes by treatment line before the last relapse; (**b**) distribution of number of therapeutic groups (single, doublet, triplet, or four or more agents) by treatment line before the last relapse; (**c**) distribution of routes of administration (oral, subcutaneous, IV, or combinations) by treatment line before the last relapse.

Figure 4 provides an overall summary diagram of the distributions observed throughout the treatment lines for therapeutic classes, number of therapeutic groups, and routes of administration before the last relapse.

The tables (left panels) represent percentages of patients and are color-coded such that green indicates an increase from the previous line, red indicates a decrease, and white indicates no change. The charts (right panels) are 100% stacked area graphs representing the relative proportion of each category in lines 1, 2, and 3+.

#### 3.3.2. At Study Appointment–After Last Relapse

**Figure 5:**
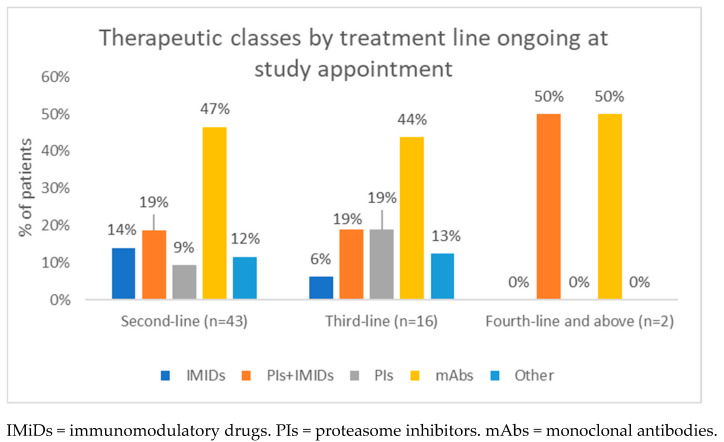
Distribution of therapeutic classes ongoing at study appointment by treatment line.

At the study appointment, all patients, except for one, were undergoing treatment (n = 61). For 91.8% of patients, the ongoing treatment regimen coincided with the one used to treat the last relapse. The median duration of treatment ongoing at study appointment was 43 days (IQR: 21–106), while median disease duration was 1.59 years (IQR: 0.96–2.82). In addition, most patients (70.5%) were receiving second-line treatment while 26.2% were undergoing third-line treatment. mAbs emerged as the leading agent during second- (46.5%) and third-line (43.8%) treatments, followed by a PIs plus IMiDs combination (18.6% and 18.8%, respectively), as shown in Figure 5.

**Figure 6:**
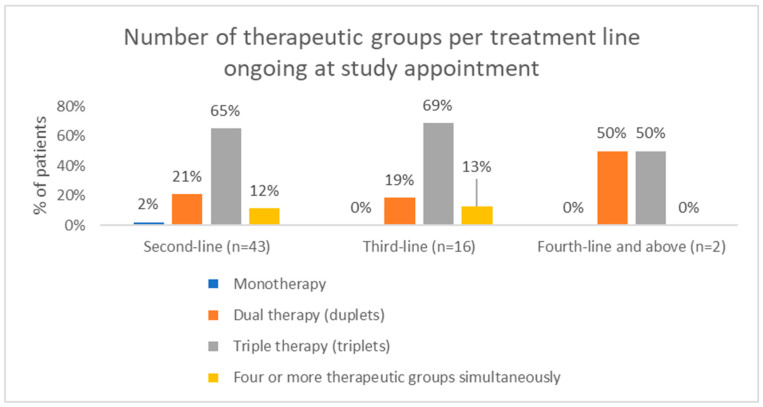
Number of therapeutic groups ongoing at study appointment by treatment line.

Triplet regimens were the most common in all treatment lines (65.1%, 68.8%, and 50% in first, second, and third and subsequent treatment lines, respectively), followed by duplet regimens (20.9%, 18.8%, and 50%, respectively) (Figure 6).

**Figure 7:**
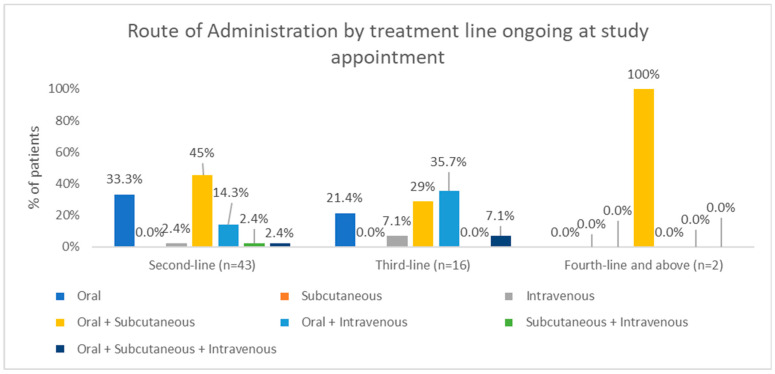
Distribution of routes of administration for treatments ongoing at study appointment by treatment line.

Oral alone and oral plus subcutaneous routes of administration were the most common in second-line and fourth-line and subsequent treatments (45.2% and 100%, respectively), while oral plus intravenous (36.4%) was the most common route in third-line treatments (Figure 7).

**Figure 8:**
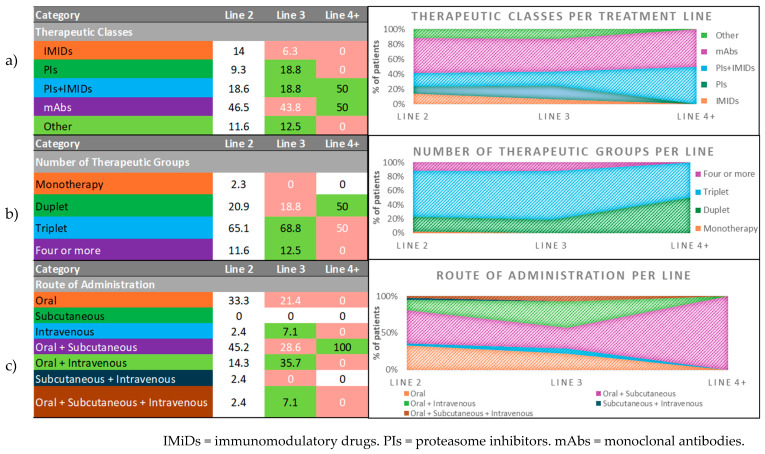
(**a**) Distribution of therapeutic classes by treatment line at study appointment; (**b**) distribution of number of therapeutic groups (single, doublet, triplet, or four or more agents) by treatment line at study appointment; (**c**) distribution of routes of administration (oral, subcutaneous, IV, or combinations) by treatment line at study appointment.

Figure 8 provides an overall summary diagram of the distributions observed throughout the treatment lines for therapeutic classes, number of therapeutic groups, and routes of administration at the last relapse (ongoing at study appointment).

The tables (left panels) represent percentages of patients and are color-coded, with green indicating an increase from the previous line, red indicating a decrease, and white indicating no change. The charts (right panels) are 100% stacked area graphs representing the relative proportion of each category in treatment lines 2, 3, and 4+.

## 4. Discussion

This analysis of rrMM patients in Portugal has provided valuable information regarding the clinical presentation of the disease, the sociodemographic characteristics of patients and treatment patterns over time.

The patient population was heterogeneous, reflecting the diverse sociodemographic profile of individuals living with rrMM. There was a predominance of the elderly, male patients, and a retired population, which was in alignment with previous research [23]. Regardless, this sample was slightly younger, with a better educational profile and with a higher rate of active employment [23].

Patient dependence negatively affects daily activities of MM patients and caregivers and access to healthcare, potentially contributing to a lack of follow-up that could ultimately contribute to the development of clinical relapses. It is of note that despite being an old and at higher risk of clinical complications population, and that around 90% of patients lived with their families, half of the patients reported being autonomous. This is aligned with previous research in Portugal that suggested a low burden of rrMM for caregivers [6]. Not surprisingly, most patients had comorbidities, such as diabetes, cardiovascular disease, and peripheral neuropathy. This highlights the multifaceted nature of the health challenges faced by these patients, adding to the complexity of managing these often poly-medicated populations. These insights underscore the importance of a broad and personalized approach to treatment that should consider not only the specific characteristics of the diseases but also individual circumstances and social backgrounds [8]. Lastly, the importance of financial constraints should not be underestimated. Although only roughly 12% of our sample needed financial assistance, research has shown that reducing costs can improve treatment compliance [25], particularly as the economic burden increases in later lines of treatment [26]. It should also be considered that the sample collected in this study was identified in the public healthcare setting, which provides unrestricted and mostly free medical care to MM patients, which may have reduced the impact of financial constraints.

Most patients enrolled in the study exhibited ISS stage II or ISS stage III at the time of diagnosis, indicating a population with an unfavorable prognosis. The cytogenetic risk assessment is an essential surrogate for understating patient prognosis and for optimizing the clinical management of the rrMM patients [10]. We found a high proportion of patients with high-risk cytogenetics. This high proportion was to be expected given that the SMART V3.0 risk classification considers d (17p), t (4;14), t (14;16), t (14;20), g (1q), and p53 mutations to all be high-risk, and a combination of these proportions of cytogenetic abnormalities in MM patients have been shown to have very high g (1q)/Amp1q counts of between 30 and 40% alone, which is expected to be even higher in rrMM patients [27,28,29]. However, it is noteworthy that cytogenetic assay data were only available for approximately one-third of the sample, a limitation that can be justified by the retrospective nature of the study [30].

The study’s description of treatment patterns revealed the changing landscape of therapeutic strategies over the last few years, as well as demonstrating the diverse array of therapeutic options in this clinical setting in Portugal, which is corroborated by previous research [2]. Commonly, therapeutic protocols include the adoption of treatment combinations, especially triplet regimens. This study showed that triplet regimens were predominant across all treatment lines. Tailoring treatments to patient profiles is essential to improve outcomes. Given the expanding arsenal of therapeutics [5,11,12,21,31,32], treatment guidelines should reflect patient-related factors as well as the biological heterogeneity of MM. Notably, this study showed that monoclonal antibodies were increasingly adopted as the number of treatment lines progressed, reflecting the integration of novel agents into the MM treatment paradigm. Conversely, PIs and IMIDs are widely adopted throughout the disease course, with a higher rate of adoption in first-line treatment. The pattern of treatment regimens used on the onset of disease relapse reveals the complexity of the therapeutic decisions in this context, which seem to be influenced by the combinations used and number of previous lines of treatment.

Despite the substantial advances in the management of rrMM, including the integration of immunotherapy, there remains a critical need for the development of novel effective and better tolerated approaches with sustained high-quality responses that delay the resurgence of relapses. This necessity is especially pronounced among high-risk patients, who face a very poor prognosis [21].

Furthermore, the analysis of treatment administration routes also revealed a notable trend. Oral alone and the combined oral + subcutaneous administrations were most common in the second, fourth, and subsequent lines of treatment, while the more classical combination of oral + intravenous administration was more common in third-line treatment. These findings highlight the adaptability towards patient-centric approaches, enabling a tailored response to the specific needs and preferences of patients, many of them residing far from the hospital, some with mobility restrictions and some depending on relatives to go to the hospital, as previously highlighted [33].

The results also support previous research, showing a trend in the increase in advanced treatment lines [34].

### 4.1. Study Limitations

The pandemic restrictions negatively impacted recruitment leading to a significant reduction in sample size and resulting in the potential introduction of selection bias. This factor makes the generalization of results to the overall MM population challenging. However, the consecutive sampling approach was devised to control for such bias, as all the patients that fulfilled the study criteria were invited to participate in the study at a routine medical appointment. Another notable limitation was the presence of missing data, especially regarding cytogenetic analysis, as already discussed. It is important to highlight that these data reflect the standard of care (SoC) at study inclusion for patients diagnosed within the previous 5 years and, therefore, may not accurately represent recent changes in SoC, namely in the context of first-line treatments.

### 4.2. Final Remarks

Given the increasing importance of patient-centric approaches in the clinical management of blood malignancies [35], the patient-reported outcomes (quality of life) collected in the CharisMMa study must be explored in future publications and research, along with a patient’s preferences and needs.

In conclusion, the findings from this study provide an overview of the MM landscape in Portugal, encompassing demographic characteristics, treatment patterns, and administration routes. Around 80% of the patients are retired or temporarily/permanently disabled and most have a low education level, which is expected in an elderly population in Portugal. Although the majority of patients enrolled in the study present with an unfavorable prognosis and comorbidities, most of them are independent or present grade I dependence, which is in line with previous research in Portugal that suggested a low burden of rrMM patients for caregivers [6]. This is an essential topic for the National Health System, when planning healthcare in a geriatric population with an oncological disease. The insights of this study may contribute to the expanding knowledge in the field of rrMM and are expected to provide enriching information about the clinical burden of this disease and serve as a valuable resource for ongoing efforts at improving the quality of care to MM patients.

As for clinical implications, as rrMM patients progress through multiple lines of therapy, the increased frailty, comorbidities, and treatment-related toxicities make less invasive and more convenient administration routes preferable. Oral therapies reduce the need for frequent hospital visits, which is beneficial for patients with mobility issues, economic limitations, or those living far from treatment centers. Also, newer-generation oral agents are becoming more widely available, expanding the treatment arsenal beyond intravenous or subcutaneous regimens. Lastly, patient preference and quality-of-life should be considered, as oral therapies offer greater flexibility and may improve adherence compared to other more invasive routes of administration, as well as healthcare resource utilization, in which outpatient treatment models and resource optimization may contribute to increasing the options for oral agents in later treatment lines.

Future research should focus on key areas to address the evolving landscape of rrMM treatment. Given that data collection occurred in Portugal between July 2020 and September 2022, new studies should explore how newer therapies—bispecific antibodies, CAR T cell therapies, and next-generation targeted agents—impact treatment patterns and patient outcomes in real-world settings. Additionally, cost-effectiveness analyses comparing different therapeutic regimens should contribute to identifying the financial burden of treatment and offer recommendations on improving the Portuguese NHS sustainability. Further exploration on how route of administration influences patient quality of life, treatment adherence, and healthcare resource utilization would also be relevant, particularly regarding the role of oral versus more invasive therapeutics. Moreover, assessing the long-term impact of treatment sequencing, including how earlier exposure to novel agents affects subsequent therapeutic options and survival outcomes, would also be pertinent. Finally, patient-reported outcomes should be included and considered to better understand treatment tolerability, symptom burden, and the psychosocial aspects of disease management, especially in rrMM patients.

## Figures and Tables

**Table 1:** Sociodemographic characteristics of rrMM patients.

Characteristics *	Total (n = 62) **
Sex	
Male	34 (54.8%)
Female	28 (45.2%)
Body Mass Index (kg/m^2^), mean (SD)	25.48 (3.86)
Area of residence	
Rural	20 (32.3%)
Urban	42 (67.7%)
Educational level (n = 58)	
Primary education	38 (65.5%)
Secondary education	12 (20.7%)
Higher education	8 (13.8%)
Employment status (n = 61)	
Unemployed	1 (1.6%)
In active employment	11 (18.0%)
Temporarily/permanently disabled	10 (16.4%)
Retired	38 (62.3%)
Other	1 (1.6%)
Cohabitation	
Living alone ^1^	4 (6.5%)
Living with relatives	56 (90.3%)
Living alone with help from a caregiver	2 (3.2%)
Need for financial assistance (yes) (n = 60)	11 (18.3%)
Degree of dependence (n = 61) ^2^	
Independent	30 (49.2%)
Dependent Grade I	15 (24.6%)
Dependent Grade II	11 (18.0%)
Dependent Grade III	5 (8.2%)
Physical activity (n = 60) ^3^	
High	1 (1.7%)
Moderate/low	34 (56.7%)
Inactive	25 (41.7%)
Currently smoking	7 (11.3%)
Alcohol consumption	5 (8.1%)

* n (%). ** n = 62 unless otherwise noted. ^1^ All patients living alone with the help of a caregiver (n = 2) required daily assistance. ^2^ Grade I (i.e., the patient needs assistance once a day to perform daily activities (DAs)), grade II (i.e., the patient needs assistance > once a day to perform DAs, but does not require constant support), and grade III (i.e., the patient needs constant support because of a lack of autonomy in the physical, mental, or sensorial area). ^3^ High (i.e., playing a sport or doing intensive exercise) and moderate/low (i.e., brisk walking and other activities such as gardening, dancing, domestic work, etc.).

**Table 2:** Patients’ clinical characteristics at diagnosis and at last relapse.

Characteristics *	(n = 62)
Age at diagnosis (years), mean (SD)	65.76 (9.24)
R-ISS stage at MM diagnosis (n = 46)	
I (Low-risk)	6 (13.0%)
II	32 (69.6%)
III (High-risk)	8 (17.4%)
Missing	16
ISS stage at MM diagnosis	
I (Low-risk)	14 (22.6%)
II	23 (37.1%)
III (High-risk)	25 (40.3%)
ECOG at MM diagnosis	
ECOG 0	22 (37.3%)
ECOG 1	22 (37.3%)
ECOG 2	12 (20.3%)
ECOG 3	3 (5.1%)
SliM CRAB ^1^ signs at last relapse	61
Plasma cell bone marrow infiltration ≥ 60%	4 (6.6%)
Serum FLC ratio ≥ 100	18 (29.5%)
>1 focal lesion visible on MRI ≥ 5 mm	5 (8.2%)
Increased blood calcium	7 (11.5%)
Renal failure	13 (21.3%)
Anemia	28 (45.9%)
Bone lesions	36 (59.0%)
Missing	1
Cytogenetic analysis at last relapse (n = 24)	
Cytogenetic abnormalities—Yes	11 (45.8%)
Cytogenetic risk ^2^	
High	8 (72.7%)
Missing	38
Clinical presentation at last relapse	
Bone fractures (n = 62)	14 (22.6%)
Plasmacytomas (at least one)—medullary/extramedullary (n = 37)	24 (64.9%)
Paraprotein levels in the blood serum (g/L), mean (SD)	53.19 (105.42)
LDH (U/I) at last relapse (n = 57), mean (SD) Normal LDH level (≤250 IU/L), n (%)	233 (169) 42 (73.7%)
Elevated LDH level (>250 IU/L), n (%)	15 (26.3%)
Free light chain concentration (g/L, serum) at last relapse:	
Kappa (g/L) (n = 30) mean (SD)	42.08 (123.0)
Lambda (g/L) (n = 19) mean (SD)	16.08 (31.8)
Heavy Chain (Yes) at diagnosis, n (%) (n = 60)	48 (80%)
IgA, n (%)	13 (21.7%)
IgD, n (%)	1 (1.7%)
IgG, n (%)	34 (56.7%)
Comorbidities at last relapse	39 (62.9%)

* n (%) ^1^ S for 60% plasma cells, Li for light chains, M for MRI lesions, C for hypercalcemia, R for renal damage, A for anemia, and B for bone disease. ^2^ Standard risk: Trisomy, t (11;14); t (6;14), High risk: d (17p), t (4;14), t (14;16), t (14;20), g (1q), and p53 mutation. Risk based on SMART V3.0. Missing values = 3. Not all patients completed the cytogenetic risk. SD—standard deviation; MM—multiple myeloma, LDH—lactate dehydrogenase, FLC—free light chain; MRI—magnetic resonance imaging.

## Data Availability

Supporting raw data and derived variables were submitted to MDPI to assist in the peer review process and provide full transparency on the results reported. The raw data supporting the conclusions of this article will be made available by the authors on request.

## Supplementary Materials

The following supporting information can be downloaded at: https://www.mdpi.com/article/10.3390/hematolrep18030034/s1. Table S1: Local IEC/IRB for each participating site.
