# Supplementary material for: Sociodemographic, Clinical, and Therapeutic Characterization of Multiple Myeloma Patients (CharisMMa Study) with Symptomatic Relapse and/or Refractory Disease: An Observational, Multicenter Study in Portugal
Source: Hematol Rep. 2026 May 19;18(3):34. doi: 10.3390/hematolrep18030034 (PMC13214959; doi:10.3390/hematolrep18030034)
Supplement: Supplementary file 1 [file hematolrep-18-00034-s001.zip › hematolrep-3385082-supplementary.pdf]

Table S1. Local IEC/IRB for each participating site

SITE#	SITE ACRONYM	RESEARCH SITE	IEC/IRB/CA/CES	DEPARTMENT
1	CHUSJ	Centro Hospitalar Universitário de São João, E.P.E.	Comissão de Ética para a Saúde do Centro Hospitalar Universitário de São João / Faculdade de Medicina da Universidade do Porto	Hematologia Clínica
2	CHULN	Centro Hospitalar Universitário de Lisboa Norte, E.P.E.	Comissão de Ética do CAML	Hematologia e Transplantação de Medula Óssea
3	IPO-Lisboa	Instituto Português de Oncologia de Lisboa Fancisco Gentil, E.P.E.	Comissão de Ética para a Saúde do IPOLFG	Hematologia
4	CHUP	Centro Hospitalar Universitário do Porto, E.P.E.	Comissão de Ética CHUP/ICBAS	Hematologia Clínica
5	CHUC	Centro Hospitalar Universitário de Coimbra, E.P.E.	Comissão de Ética para a Saúde do CHUC, E.P.E	Hematologia
6	CHVNG-E	Centro Hospitalar de Vila Nova de Gaia/Espinho E.P.E.	Comissão de Ética para a Saúde do CHVNG-E	Hematologia
7	IPO-Porto	Instituto Português de Oncologia do Porto Fancisco Gentil, E.P.E.	Comissão de Ética para a Saúde do IPO Porto EPE	Onco-Hematologia
8	HBraga	Hospital de Braga, E.P.E.	Comissão de Ética do Hospital de Braga (CEHB)	Oncologia Médica
